# Etiopathogenesis of Sheehan's Syndrome: Roles of Coagulation Factors and TNF-Alpha

**DOI:** 10.1155/2014/514891

**Published:** 2014-04-10

**Authors:** Halit Diri, Elif Funda Sener, Fahri Bayram, Nazife Tascioglu, Yasin Simsek, Munis Dundar

**Affiliations:** ^1^Department of Endocrinology, Erciyes University Medical School, 38039 Kayseri, Turkey; ^2^Department of Medical Biology, Erciyes University Medical School, 38039 Kayseri, Turkey; ^3^Department of Medical Genetics, Erciyes University Medical School, 38039 Kayseri, Turkey

## Abstract

Sheehan's Syndrome (SS) is defined as pituitary hormone deficiency due to ischemic infarction of the pituitary gland as a result of massive postpartum uterine hemorrhage. Herein, we aimed to investigate the roles of *Factor II* (G20210A), *Factor V* (G1691A), *MTHFR* (C677T and A1298C), *PAI-1* 4G/5G, and *TNF-**α*** (−308  G > A) gene polymorphisms in the etiopathogenesis of SS. Venous blood samples were obtained from 53 cases with SS and 43 healthy women. Standard methods were used to extract the genomic DNAs. *Factor II* (G20210A), *Factor V* (G1691A), and *MTHFR* (C677T and A1298C) polymorphisms were identified by real-time PCR. *PAI-1* 4G/5G and *TNF-**α*** (−308  G > A) gene polymorphisms were detected with polymerase chain reaction (PCR) and restriction fragment length polymorphism (RFLP) methods. According to statistical analysis, none of the polymorphisms were found to be significantly higher in the SS group compared to the control group. Hence, we suggest that genetic factors other than *Factor II, Factor V, MTHFR, PAI-1*, and *TNF-**α*** gene polymorphisms should be researched in the etiopathogenesis of SS.

## 1. Introduction

Sheehan's Syndrome (SS) is defined as pituitary hormone deficiency due to ischemic infarction of the pituitary gland as a result of massive postpartum uterine hemorrhage [1]. Although the onset of SS can involve acute severe panhypopituitarism in some patients, the majority of SS patients are diagnosed with a clinically subtle partial pituitary deficiency and therefore their diagnoses and treatments are delayed for many years [2]. The prevalence of SS is not clearly known, presumably due to the great number of nondiagnosed patients. It is a rarely encountered disorder in developed countries due to good obstetric care [3, 4]. However, its prevalence is estimated to be still high in developing countries in which many deliveries take place at home [5]. Zargar et al. estimated the prevalence of SS to be 3.1% among women in India, and about two-thirds of those women had given birth at home [6].

As the etiopathogenesis of SS is not clear, disorders of coagulation have been investigated in some studies. It has been reported that disseminated intravascular coagulation (DIC) can cause postpartum hypopituitarism [7, 8]. In addition, Cakir et al. found protein S deficiency in 2 out of 12 patients with SS [9]. Importantly, in another study, Gokalp et al. investigated inherited hypercoagulation as a risk factor of SS. They found that frequency of* MTHFR (methylenetetrahydrofolate reductase) *C677T and* MTHFR* A1298C polymorphisms was significantly higher among their 38 patients with SS compared to the healthy control group [10]. In addition,* Factor II* (G20210A),* Factor V* (G1691A), and* PAI-1* 4G/5G mutations were also more common among the SS patients, but no significant differences were observed.

Our aim in this study was to investigate gene polymorphisms of* MTHFR* C677T and A1298C,* Factor II* (G20210A),* Factor V* (G1691A), and* PAI-1 (plasminogen activator inhibitor-1) *4G/5G that are associated with inherited hypercoagulation and* TNF-*α* (tumor necrosis factor-alpha) *308 G > A that is associated with apoptosis of pituitary cells due to autoimmunity. Knowing the frequencies of these polymorphisms among SS patients will be helpful in understanding their roles in the etiopathogenesis of SS.

## 2. Patients and Methods

### 2.1. Study Design

Fifty-three patients who were previously diagnosed with SS and 43 healthy women were enrolled in this study which was conducted between 2011 and 2013. The blood samples of patients with SS who were followed up by the Endocrinology Department of Erciyes University Medical School were collected following 12 hours of fasting. The healthy female volunteers in the control group were chosen from hospital staff and their relatives. Written informed consent was obtained from all participants before registering them for the study.

In addition to the demographic information, the medical history and drug use of the participants were examined in detail. The most important inclusion criterion for the SS group was having the exact diagnosis of SS. Therefore, it was emphasized that all of the following criteria for diagnosis were met: (a) at least one pituitary hormone deficiency found in basal levels or via dynamic tests if required; (b) massive postpartum uterine hemorrhage history at last delivery; (c) agalactia and amenorrhea after the last delivery; (d) exclusion of all other causes of pituitary deficiency; (e) observation of partial or complete empty sella on magnetic resonance imaging (MRI). Exclusion criteria for SS patients were having comorbidity or being on other treatments than glucocorticoid and thyroid hormone replacement therapies which were adequately performed. In order to avoid the effects of replacement therapies of growth hormone (GH) and gonadal steroids on genotyping studies, such treatments were stopped 3 months before the blood collection for the genetic analyses. In addition, women in the control group did not have any histories of disease and were not on any drug therapies.

### 2.2. Hormonal Analyses

Basal levels of hormones were analyzed to determine hypopituitarism in all patients. In addition, the insulin tolerance test or glucagon stimulation test was performed to identify growth hormone (GH) deficiency and secondary adrenal failure. Basal hormone levels including free T4 (normal: 0.88–1.72 ng/dL), thyroid stimulating hormone (TSH; normal: 0.57–5.6 mIU/mL), adrenocorticotropic hormone (ACTH; normal: 0–46 pg/mL), cortisol (normal: 9–23 *μ*g/dL), prolactin (PRL; normal for postmenopausal women: 2.4–29.8, and premenopausal women: 3.3–29.8 ng/mL), follicle stimulating hormone (FSH; normal for postmenopausal women: 23.9–119.1, and premenopausal women: 2.0–9.8 mIU/mL), luteinizing hormone (LH; normal for postmenopausal women: 16.3–54.8, and premenopausal women: 0.7–17.3 mIU/mL), estradiol (E2; normal for postmenopausal women: 14.4–44.5, and premenopausal women: 18.9–246.7 pg/mL), and insulin-like growth factor-1 (IGF-1; reference intervals varied by age) were measured in the hormone laboratories of Erciyes University Medical School. Methods of assays and commercial kits were as follows: GH: immunoradiometric assay (IRMA), Immunotech SAS, France; IGF-1: IRMA, Immunotech SAS, France; ACTH: IRMA, Cisbio Bioassays, France; cortisol: radioimmunoassay (RIA), Immunotech s.r.o, Czech Republic; PRL, TSH, fT4, FSH, LH, estradiol: Immunoassay, Siemens Advia centaur XP-USA.

### 2.3. Genotyping Studies

2 mL venous blood samples were collected in EDTA-containing tubes for DNA analyses. Total genomic DNA was extracted by standard methods and DNA samples were stored at −20°C until the analyses of polymorphisms. Genotyping studies were conducted in Erciyes University Genome and Stem Cell Center (GENKOK). A total of 20 *μ*L PCR mixture with 5 *μ*L sample DNA was analyzed by using a LightCycler FastStart DNA Master HybProbe to perform the genotypings of* MTHFR* C677T and A1298C,* Factor II* (prothrombin) G20210A, and* Factor V* G1691A according to manufacturer's instructions (Roche Diagnostics, Germany). In addition, Roche LightCycler 480 Software was used for detecting different genotypes of these polymorphisms.

Genotypes of the* TNF-*α**-308 G/A and* PAI-1* 4G/5G polymorphisms were detected by analyses of polymerase chain reaction (PCR) and restriction fragment length polymorphism (RFLP) results.* PAI-1 *4G/5G polymorphisms were analyzed by forward primer 5′-CACAGAGAGAGTCTGGCCACGT-3′ and 5′-CCAACAGAGGACTCTTGGTCT-3′ reverse primer. The genomic region of interest was amplified by using PCR for 30 cycles with a denaturation temperature of 94°C for 3 min, 94°C for 30 sec, 60°C for 30 sec, and 72°C for 30 sec. The final extension step at 72°C was extended for 1 min. Amplified 98 base pair (bp) products were digested overnight with* Bsl I* at 55°C and subjected to 4% agarose gel electrophoresis [11].

The PCR procedure was performed in a total volume of 50 *μ*L containing 5 *μ*L genomic DNA, 10x PCR buffer, dNTPs (2.5 mM), MgCl_2_ (1.5 mM), Taq DNA polymerase (1 U/mL), 5′AGGCAATAGGTTTTGAGGGCCAT-3′ forward primers, and 5′TCCTCCCTGCTCCGATTCCG-3′ reverse primers for* TNF-*α** [12]. The cycling conditions consisted of denaturation at 95°C for 5 min, followed by 30 consecutive cycles of 95°C for 1 min, 60°C for 1 min, and 72°C for 1 min, with a final elongation at 72°C for 5 min. The 107 bp PCR products were separated by electrophoresis on a 2% agarose gel and visualized with UV illumination and ethidium bromide staining. After amplification, the PCR products were digested overnight at 37°C with* Nco I* restriction enzyme and analyzed by 3% agarose gel electrophoresis.

### 2.4. Statistical Analyses

Statistical analyses were performed by using SPSS 15.0 (SPSS Inc., Chicago, IL). Demographic data were presented as mean ± SD. Binomial variables were analyzed using Pearson's chi-square test or Fisher's exact test. Moreover, the odds ratios (OR) were calculated with 95% confidence intervals (CI) using logistic regression analysis. Comparisons of genotype frequencies were made between patients and controls, and *P* values less than 0.05 were considered as statistically significant.

## 3. Results

Panhypopituitarism was detected in 38 (71.7%) patients with SS, while partial hypopituitarism was detected in 15 (28.3%) patients. Importantly, all patients had gonadotropin and GH deficiencies, but deficiencies of ACTH, TSH, and prolactin were not found in all of the patients (Table 1).

When comparing groups, it was revealed that there was no significant difference between the mean age of the 53 cases with SS and the 43 healthy controls, 63.2 ± 12.5 and 60.3 ± 9.3 years, respectively. In addition, no difference was detected in terms of mean body mass indexes (BMIs). The mean BMI was 28.9 ± 2.8 kg/m^2^ in the SS group and 29.2 ± 3.3 kg/m^2^ in healthy women. Moreover, the SS and healthy control groups did not significantly differ according to polymorphism ratesof* Factor II* (G20210A),* Factor V* (G1691A),* MTHFR* (C677T and A1298C),* PAI-1* (4G/5G), and* TNF-*α** (−308 G > A) genes, except for* PAI-1* (4G/5G). The* PAI-1* (4G/5G) mutation was detected in 26 (60.5%) of the 43 control cases and in 20 (37.7%) of the 53 SS cases. Frequencies and comparisons of polymorphisms are demonstrated in Table 2.

## 4. Discussion

The physiological enlargement of the pituitary gland during pregnancy plays a significant role in onset of SS, because severe bleeding does not lead to pituitary deficiency in women unless they are pregnant. Even though the pathogenesis of SS has not yet been fully clarified, the basis of its pathology has been identified as infarction and ischemic necrosis that develops due to the interruption of arterial blood flow in the anterior pituitary gland [13]. However, the cause of the interruption in the blood flow is not clear. Considered potential mechanisms are arterial thrombosis similar to that seen in stroke, development of arterial spasm as a result of severe hypotension that is due to massive uterine bleeding, or compression of pituitary vessels due to relatively small sella turcica volume associated with enlargement of the pituitary during pregnancy [14]. Furthermore, autoantibodies detected in many patients against the pituitary gland have been suggested as a contributing factor in the etiopathogenesis of SS [15].

In the etiopathogenesis of SS, our study findings did not yield a significant difference between the control and SS patient groups in terms of mutations of blood composition anomalies that lead to inherited hypercoagulation. In other words, in the SS group none of the genes had higher polymorphism rates than those in the control group. There are numerous other acquired (prolonged immobilization, pregnancy, oral contraceptive pills, advanced age, obesity, cigarette use, hypertension, etc.) or genetic (protein C or S deficiency, antithrombin-III deficiency, platelet GPIIb/IIIa HPA-Ib mutation, elevated levels of Factors VII, VIII, IX, and XII, Von Willebrand disease, fibrinogen, etc.) factors that are known to cause hypercoagulation. An important limitation of our study is that not all of the parameters associated with hypercoagulation were investigated. Therefore, it is hard to conclude, based only on our study findings, that inherited hypercoagulation is not involved in SS.

Although, the mutation rates of* MTHFR* C677T and A1298C genes show social differences, homozygous mutations are estimated to be 10%, and heterozygous mutations are about 40% [16, 17]. Approximately, 15% of the population is reported to carry both heterozygous genetic mutations together, due to their close relations [17]. Although the rates of* MTHFR* gene polymorphisms were not higher in the SS group than in the control group in our study, more important issues are whether the SS patients had vitamin B6, vitamin B12, and/or folate deficiency and thus a disorder in* MTHFR* gene expression during their last pregnancies.

The heterozygous mutations in* Factor II* (prothrombin) G20210A are encountered at a 2-3% rate among Caucasians [18]. Plasma Factor-II levels (prothrombin) increase as a result of mutations. In that case, risk of venous thrombosis is higher than arterial thrombosis risk. Venous thrombosis risk among individuals carrying this mutation is increased by 2-3 times. One study reported that coronary artery risk is increased by 1.31 times in individuals with prothrombin G20210A mutation [19].

While varying across different populations, the incidence rate of Factor V Leiden heterozygous mutation is approximately 5%, but its homozygous mutation is rare [20]. As a result of the mutation in the* Factor V* gene (G1691A), activated protein-C cannot inhibit Factor V molecules. This, in turn, results in a deterioration of the bleeding and coagulation balance in favor of coagulation. This mutation is reported to increase thrombosis risk fivefold when heterozygous, and by 10–80-fold when homozygous [21, 22].* Factor V* Leiden has also been shown to increase complications such as miscarriage, preeclampsia, and abruptio placentae by a minimum of 2-3 times [20].

*Plasminogen activator inhibitor-1 (PAI-1)* inhibits tissue* plasminogen activator (tPA) *and* urokinase* which are proteins leading to plasminogen activation and fibrinolysis. The result is inhibition of fibrinolysis. The estimated frequences of* PAI-1* 4G/4G, 4G/5G and 5G/5G are, respectively, about 35%, 50%, and 15% [23, 24]. Polymorphisms of* PAI-1* 4G/4G and 4G/5G are reported to cause an increase in PAI-1 levels and thus risk of arterial thrombosis rather than of venous thrombosis [25, 26]. Pregnant women who carry the homozygous* PAI-1* 4G/4G mutation are also known to have an increased risk of miscarriage [27]. However, there are other studies reporting that patients with ischemic stroke and coronary artery disease do not have increased PAI-1 4G/5G polymorphism [28, 29]. Interestingly, our results showed that the 4G/5G polymorphism rate was 60.5% among healthy women, while it was 37.7% among SS patients. This finding may suggest that the 4G/5G polymorphism does not always lead to thrombophilia, unless accompanied by some acquired factors.

TNF-*α* (tumor necrosis factor-alpha) is a cytokine that plays an important role in the regulations of immune functions, cell proliferation and differentiation, apoptosis due to autoimmunity, coagulation, adipocyte, lipid and glucose metabolism. The 308 G/A polymorphism of* TNF-*α** which leads to elevated TNF-*α* levels is known to have an important impact on the development of autoimmunity diseases, metabolic syndromes, cancers, and psychiatric disorders [30, 31]. Additionally, an association between high TNF-*α* levels and hypercoagulation has also been detected [32].

Autoimmunity is a potential risk factor in the development of SS and 35% of patients have been shown to have anti-pituitary autoantibodies [33]. These antibodies are presumably generated in reaction to the necrotic pituitary gland tissue found after ischemic infarction and seem to be responsible for the chronic but progressive course of SS [15]. On another note, increased estrogen levels during pregnancy make adenohypophysis cells, particularly the lactotroph cells, more vulnerable to apoptosis due to autoimmunity as a result of elevated expression of the TNF-*α* gene in rats [34]. This condition may also be associated with the vulnerability of an enlarged pituitary to ischemic necrosis at the end of pregnancy. Estrogen levels gradually increase during pregnancy and peak close to completion of the full term. In this regard, we hypothesized that autoimmunity caused by* TNF-*α** 308 G/A mutation can be a contributing factor in the etiopathogenesis of SS. However, no increase in the rate of* TNF-*α** 308 G/A polymorphism was observed among the SS patients when compared to the control group.

## 5. Conclusion

As demonstrated in our study, the disorders suggested for SS etiopathogenesis and the associations among them are highly complicated. Thrombophilia and autoimmunity in SS are also multifaceted and complicated mechanisms. However, the results of our study showed no increase in* Factor II*,* Factor V*,* MTHFR*,* PAI-1*, and* TNF-*α** gene polymorphism rates among SS patients compared to the control group. Hence, genetic factors other than these gene polymorphisms should be researched in the etiopathogenesis of SS. Clarifying SS etiopathogenesis via further studies will be particularly beneficial in identifying women susceptible to SS prior to disease development.

## Figures and Tables

**Table 1:** Distribution of deficient hormones in patients with SS.

Deficient hormones	Number and percentage of patients
FSH-LH + GH + TSH + ACTH + PRL	38 (71.7%)
FSH-LH + GH + TSH + PRL	4 (7.6%)
FSH-LH + GH + ACTH + PRL	2 (3.8%)
FSH-LH + GH + ACTH + TSH	3 (5.7%)
FSH-LH + GH + PRL	1 (1.9%)
FSH-LH + GH + TSH	4 (7.6%)
FSH-LH + GH	1 (1.9%)

Total	53 (100%)

**Table 2:** Distribution of thrombophilic and cytokine genes among Sheehan's Syndrome cases and controls.

	Sheehan's Syndrome patients (*n*: 53)		Controls (*n*: 43)		OR (95% CI)	*P* value^‡^
	*n *	(%)	*n *	(%)		
*MTHFR* C677T						
Genotype						
CC (normal)	31	58.5	25	58.1	1 (Ref)	—
CT (heterozy.)	19	35.8	13	30.2	1.179 (0.489–2.843)	0.714
TT (homozy.)	3	5.7	5	11.6	0.484 (0.105–2.224)	0.351
*P* value^†^	0.538					

*MTHFR* A1298C						
Genotype						
AA (normal)	17	32.1	15	34.9	1 (Ref)	—
AC (heterozy.)	28	52.8	19	44.2	1.3 (0.525–3.219)	0.570
CC (homozy.)	8	15.1	9	20.9	0.784 (0.241–2.549)	0.686
*P* value^†^	0.646					

*Factor II* G20210A						
Genotype						
GG (normal)	50	94.3	42	97.7	1 (Ref)	—
GA (heterozy.)	3	5.7	1	2.3	2.520 (0.253–25.136)	0.431
AA (homozy.)	—	—	—	—	—	—
*P* value^†^	0.416					

*Factor V* G1691A						
Genotype						
GG (normal)	47	88.7	37	86	1 (Ref)	—
GA (heterozy.)	5	9.4	4	9.3	0.984 (0.247–3.925)	0.982
AA (homozy.)	1	1.9	2	4.7	0.394 (0.034–4.511)	0.454
*P* value^†^	0.741					

*PAI-1* 4G/5G						
Genotype						
5G/5G (normal)	21	39.6	7	16.3	1 (Ref)	—
4G/5G (heterozy.)	20	37.7	26	60.5	0.256 (0.091–0.722)	0.01
4G/4G (homozy.)	12	22.6	10	23.3	0.4 (0.121–1.326)	0.134
*P* value^†^	0.03					

*TNF-*α** −308 G>A						
Genotype						
GG (normal)	43	81.1	34	79.1	1 (Ref)	—
GA (heterozy.)	9	17	9	20.9	0.791 (0.283–2.210)	0.654
AA (homozy.)	1	1.9	—	—	—	—
*P* value^†^	0.6					

Notes: heterozy.: heterozygous; homozy.: homozygous.

^†^Significance of *χ* ^2^ values which were obtained from a Chi-Square test.

^‡^Significance of odds ratios which were obtained from a logistic regression model.
